# The intestinal microbial composition in Greylag geese differs with steatosis induction mode: spontaneous or induced by overfeeding

**DOI:** 10.1186/s42523-020-00067-z

**Published:** 2021-01-06

**Authors:** Christelle Knudsen, Julien Arroyo, Maxime Even, Laurent Cauquil, Géraldine Pascal, Xavier Fernandez, Franck Lavigne, Stéphane Davail, Sylvie Combes, Karine Ricaud

**Affiliations:** 1 GenPhySE, Université de Toulouse, INRAE, ENVT, F-31326 Castanet Tolosan, France; 2 ASSELDOR, Station d’expérimentation appliquée et de démonstration sur l’oie et le canard, La Tour de Glane, 24420 Coulaures, France; 3 Université de Pau et des Pays de l’Adour, E2S UPPA, INRAE, NUMEA, Saint-Pée-sur- Nivelle, 64310 Pau, France

**Keywords:** *Anser anser*, Gut microbiota, Fatty liver, Migratory bird

## Abstract

**Background:**

Relationships between microbial composition and steatosis are being extensively studied in mammals, and causal relations have been evidenced. In migratory birds the liver can transiently store lipids during pre-migratory and migratory phases, but little is known about the implications of the digestive microbiota in those mechanisms. The Landaise greylag goose (*Anser anser*) is a good model to study steatosis in migratory birds as it is domesticated, but is still, from a genetic point of view, close to its wild migratory ancestor. It also has a great ingestion capacity and a good predisposition for hepatic steatosis, whether spontaneous or induced by conventional overfeeding. The conventional (overfeeding) and alternative (spontaneous steatosis induction) systems differ considerably in duration and feed intake level and previous studies have shown that aptitudes to spontaneous steatosis are very variable. The present study thus aimed to address two issues: (i) evaluate whether microbial composition differs with steatosis-inducing mode; (ii) elucidate whether a digestive microbial signature could be associated with variable aptitudes to spontaneous liver steatosis.

**Results:**

Performances, biochemical composition of the livers and microbiota differed considerably in response to steatosis stimulation. We namely identified the genus *Romboutsia* to be overrepresented in birds developing a spontaneous steatosis in comparison to those submitted to conventional overfeeding while the genera *Ralstonia*, *Variovorax* and *Sphingomonas* were underrepresented only in birds that did not develop a spontaneous steatosis compared to conventionally overfed ones, birds developing a spontaneous steatosis having intermediate values. Secondly, no overall differences in microbial composition were evidenced in association with variable aptitudes to spontaneous steatosis, although one OTU, belonging to the *Lactobacillus* genus, was overrepresented in birds having developed a spontaneous steatosis compared to those that had not.

**Conclusions:**

Our study is the first to evaluate the intestinal microbial composition in association with steatosis, whether spontaneous or induced by overfeeding, in geese. Steatosis induction modes were associated with distinct digestive microbial compositions. However, unlike what can be observed in mammals, no clear microbial signature associated with spontaneous steatosis level was identified.

## Background

In birds, unlike mammals, the liver represents the main lipid synthesis site and can transiently store lipids, as it is the case in migratory birds during pre-migratory and migratory phases although storage mainly occurs in peripheral adipose tissues [1, 2]. During the pre-migratory phase increased feed intake is the main contributor to overall fattening [3], but increased digestive efficiency has also been observed, although variable between species [4].

The Landaise greylag goose (*Anser anser*) is a good model to study steatosis in migratory birds as it is domesticated, and can therefore be easily bred in experimental conditions, but is still, from a genetic point of view, close to its wild migratory ancestor [5]. It also has a great ingestion capacity and a good predisposition for hepatic steatosis, whether spontaneous [6] or induced by conventional overfeeding [7]. However, this last practice is highly questioned today for animal welfare reasons. Research is therefore being conducted to seek for alternative and more ethical methods without overfeeding to produce waterfowl fatty liver. In experimental conditions, spontaneous steatosis has been induced [6] by mimicking pre-migratory conditions, performing trials during winter season with a reduction in day length and alternations in food availability with a restricted access to a pellet diet followed by 12 weeks of ad libitum corn feeding. However, rearing time, and consequently overall feed intake and environmental impacts, are drastically increased with a liver weight that is halved compared to the conventional system based on overfeeding [8]. Previous studies using this experimental setup have also evidenced high variability in the aptitude to spontaneous steatosis (45–65% in variability coefficient with an average liver weight increase from around 90 g to 500 g) that can in part be explained by the inter-individual variability in feed intake [6, 9]. Other parameters such as genetics and the digestive microbiota could however contribute to this variability. Indeed, in mammals growing evidence suggests that alterations in the digestive microbiota can contribute to the onset and progression of Non Alcoholic Fatty Liver Disease (NAFLD), although diet plays the main role in the etiology of this disease (for review, [10]). The digestive microbiota could then be another contributing factor to the variable aptitude to spontaneous steatosis but also a potential actor on feeding behavior [11] in geese.

Few studies have assessed associations between the microbiota and steatosis in greylag geese, or migratory birds. In waterfowl, studies have focused on overfed ducks [12–14] and geese [15]. However, overfeeding differs greatly from spontaneous steatosis induction in duration (16 days vs 12 weeks in geese), daily feed intake level and average liver weight (1000 vs 500 g), variability (20 vs 45–60%) [16] and chemical composition [17]. Also, in conventional overfeeding, feed intake being controlled, no impact of the digestive microbiota on feed intake can be evaluated. The present study thus aimed to address two issues. First, as the conventional (overfeeding) and alternative (spontaneous steatosis induction) systems differ considerably, we wanted to evaluate whether microbial composition differed with steatosis-inducing mode. Second, we wanted to elucidate whether the digestive microbiota could be correlated with variable aptitudes to spontaneous liver steatosis.

## Methods

The in vivo experiment was performed between July 2015 and February 2016 at the Goose and Duck Breeding Station (Coulaures, France; experimental approval A24–137-1). Technical staff and scientists all had individual authorizations to conduct animal experimentation in accordance with good animal practices issued by the DDCSPP (Direction Départementale de la Cohésion Sociale et de la Protection des Populations) and slaughter was performed according to the European Council regulations [18]. All diets used during the rearing period met the National Research Council’s (NRC) requirements [19] and were manufactured by Sanders Périgord (Boulazac, Dordogne, France).

### Feeding programs and housing management

Eighty five male 43-day-old Greylag Landaise geese (Maxipalm®; *Anser anser*) were reared in housing, feeding and management conditions as previously described [20] given a grower-finisher diet (Apparent metabolizable energy corrected for nitrogen (AMEn) 11.5 MJ/kg, Crude Protein (CP) 161 g/kg) ad libitum until 56 days of age. After that, access to the grower finisher diet was limited to 2 to 3 h/day.

**Fig. 1:**
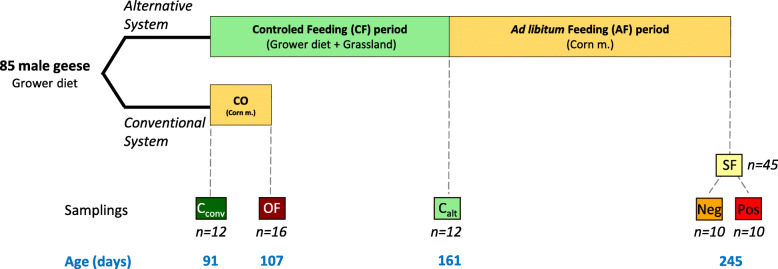
Experimental design. Two steatosis induction rearing systems were studied, the conventional one using conventional overfeeding (CO) with a corn mixture (Corn m.) and an alternative one using a spontaneous steatosis stimulation alternating a controlled feeding (CF) period with a grower diet and an access to a grassland and an Ad libitum feeding (AF) period with the same corn mixture as in the conventional system. In both systems samplings were made before (C_conv_ and C_alt_ groups) and after corn mixture feeding (OF and SF groups). At the end of the AF period 45 geese were sampled and the 10 animals with the highest (PosSF) and the lowest (NegSF) liver weights were selected. AF: Ad libitum feeding, C: Control, CF: Controled Feeding, CO: Conventional overfeeding, OF: Overfeeding group, SF: Spontaneous fattening groups

At 91 days of age, birds were separated in two experimental systems, a “conventional” system (Conv., *n* = 28 birds) based on overfeeding and an “alternative” system (Alt., *n* = 57 birds) (Fig. 1), and body weight (BW) at 91 days was standardized between the two systems. In the conventional system, birds were overfed from 91 to 107 days of age in housing, feeding and management conditions previously described by Arroyo et al. [20]. Birds were given a corn mixture composed of 34% of corn flour, 24% of whole corn, 40% of water, and 2% of vitamins. Birds in the alternative system were housed in a building of 73m^2^ and submitted to a breeding program adapted from the protocol of Guy et al. [6] in order to mimic pre-migratory conditions. Briefly, birds were submitted to a controlled feeding period (CF) with the commercial grower-finisher diet used in the previous period (280 g/day from 91 to 133 days of age and 180 g/day from 133 to 161 days of age). It aimed to ensure normal growth, prevent excessive fattening, increase ingestion capacity and induce hyperphagia when feed was subsequently provided ad libitum. As geese are herbivorous birds [21], in order to maintain a large crop volume and further improve the ingestion capacity, birds had free access to a grassland of 4350 m^2^. This was an innovation compared to previous studies on spontaneous steatosis stimulation where no access to an outdoor grassland was provided and animals were housed in a closed building from 105 days onwards [6, 9, 22]. As a result, no artificial light stimulation was performed. Birds were however submitted to the natural reduction in day length from 13h16min at 91 days of age (Early September) to 9h45min at 161 days of age (Mid November). At the end of the CF period the access to the outdoor area was suppressed and the birds were fed freely with the same corn mixture as in the conventional system for 12 weeks (Ad libitum feeding (AF) period, 161 to 245 days of age at the end of which day length was of 9h53min (Early February)). We chose to use the corn mixture as feed in order to have equivalent diets provided to birds in both systems (Conventional vs Alternative) during steatosis induction. Ambient temperatures ranged from + 4 °C to + 16 °C along the AF period and were on average of + 9 °C (Supplemental Fig. 1).

### Measurements, slaughter and samplings

Feed intake was recorded individually at each meal during overfeeding in the conventional system and per pen, daily, during the CF period and weekly during the AF period, in the alternative system. During the CF period refusals were equal to zero. Corn mixture intake is expressed in g of DM/bird (estimating DM_whole corn_ = 0.89) while the Grower-Finisher diet intake is expressed in g/bird. Crop volume was measured before slaughter according to the procedure described by Arroyo et al. [23]. Animals were weighed at slaughter.

In both systems, birds were slaughtered before steatosis induction (91 days of age in the conventional system, “C_conv_” group, *n* = 12 and at 161 days of age in the alternative system, “C_alt_” group, *n =* 12) and after steatosis induction at 107 days of age, after conventional overfeeding (CO), in the conventional system (Overfed, “OF” group, *n* = 16) and at 245 days of age, after 12 weeks of Ad libitum feeding (AF), in the alternative system (Spontaneous fattening, “SF” group, *n* = 45) (Fig. 1). Birds were electrically stunned and immediately killed by an exsanguination through a ventral cut of neck blood vessels. The liver and abdominal fat were carefully removed and weighted. Immediately after evisceration, ileal content was sampled and stored at − 80 °C until further analyses. The right lobe of the liver was sampled, immediately frozen in liquid nitrogen and stored at − 80 °C until further analyses.

In the SF group liver weight was of 158 ± 69 g. Two subsets of 10 geese from the SF group were generated: PosSF, the 10 geese with the highest liver weights (> 200 g) and NegSF, the 10 geese with the lowest liver weights (< 100 g) (Supplemental Fig. 2A). Solely these two subsets were analyzed and will be presented for the SF group.

### Biochemical characterization of the livers

Dry matter (DM) content was determined by drying grinded fresh liver samples in an oven at 105 °C for 24 h and mineral matter determined after 16 h at 550 °C. As lipid contents were high, samples were mixed with fine sand to increase the exchange surface and avoid fat crust formations. Total nitrogenous matter was determined after total combustion by the Dumas method [24] using a Leco auto-analyser (FP 428; Leco Corp., St Joseph, Michigan, USA). Total lipid content was determined after cold extraction in a chloroform/methanol mixture (2/1, V/v) and measured gravimetrically according to the method described by Folch et al. [25], with 1 g of fresh liver for 50 ml of extraction volume. For measurements of lactate, glucose and glycogen, 2 g of liver were homogenized with an Ultra Turrax (IKA T-25, Fisher Scientific, Illkirch, France) in 10 ml of 0.5 M perchloric acid. After a 15 min centrifugation at 2500 g at 4 °C, supernatants were used for glucose determination before (total glucose) and after (free glucose) hydrolyzation of glycogen with amyloglucosidase according to the method of Dalrymple and Hamm [26]. Glycogen was calculated as the difference between total and free glucose. Lactate level was determined on the same supernatants through an absorbance measurement at 340 nm after addition of lactate dehydrogenase, according to the method of Bergmeyer et al. [27]. Glucose, glycogen and lactate levels are expressed in μmol per gram of fresh liver.

### DNA extractions, 16S rRNA gene sequencing and sequence analysis

Ileal content DNA was extracted combining mechanical, chemical and thermic lysis with an Ultra Turrax Digital Homogenizer (IKA T-25, Fisher Scientific, Illkirch, FR) and the QIAamp Fast DNA Stool Mini Kit (Qiagen Gmbh, Hilden, DE) according to the manufacturer’s instructions. Quantity and quality of extracted DNA were determined with a NanoVue Plus Spectrophotometer (GE Healthcare, Vélizy-Villacoublay, FR). The V3-V4 regions of 16S rRNA genes were amplified by PCR and sequenced by MiSeq Illumina Sequencing at the Genomic and Transcriptomic Platform (GeT-PlaGe, INRAE, Toulouse, France).

16S rRNA gene amplicon sequences were analyzed using the FROGS pipeline according to standard operating procedures [28]. Only amplicons with size between 380 and 500 bp, without ambiguous bases and with the two primers were kept. Due to low number of reads one sample of the C_alt_ group (2236 reads) and one of the NegSF group (3040 reads) were excluded from further analyses. Sequences were clustered (d = 1 + d = 3) using Swarm and chimeras were removed. The remaining clusters were filtered. Clusters that were present in at least 7 samples and with abundances greater than 0.005% of total sequences [29] were kept as OTUs (operational taxonomic units). Taxonomic affiliation of OTUs was obtained using the Silva132 database with a minimum pintail quality of 80 [30]. Samples had on average 28,015 ± 8698 reads (min: 5586, max: 46184) with 266 OTUs represented (98 ± 28 OTUs per sample).

### Statistical analyses

Statistical analyses were performed using R software version 4.0.2. The microbial composition was analysed with the phyloseq package [31]. For alpha and beta diversity analyses, samples were rarified to even depth (5586 reads). Beta-diversity was determined using the Bray-Curtis dissimilarity method and plotted by non-Metric Dimensional Scaling (nMDS) ordination method. To assess group differences an ADONIS pairwise test with the Bray-Curtis distance was carried out. A Partial Least Squares Discriminant Analyzis (PLS-DA) was performed to determine the most discriminant OTUs separating NegSF and PosSF geese using the R mixOmics package [32]. Taxa differential abundance analyses and PLS-DA were performed on unrarefied data with OTUs with abundances above 0.1% of total sequences in at least one experimental group, which was considered as a quantitative threshold.

Growth and slaughter performances, biochemical parameters, microbial alpha diversity and relative abundances at phylum, family, genus and OTU levels were analysed using a non-parametric Kruskal-Wallis test with the experimental group as factor. When significant (*P* < 0.05), groups were compared pairwise with a Wilcoxon test. For taxa relative abundances *p*-values were adjusted for multiple tests with the false discovery rate (FDR) method.

## Results

### Growth, intake and performances at slaughter

**Fig. 2:**
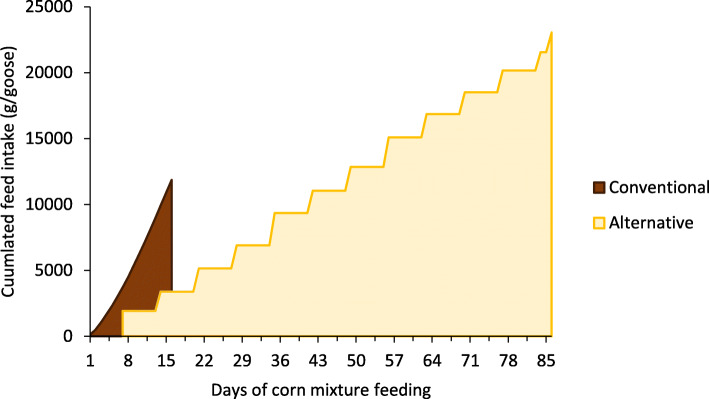
Cumulated feed intake during the corn mixture feeding periods (CO during 16 days in the Conventional system and AF during 12 weeks in the alternative system). Feed intake is expressed in equivalent of dry matter. Intake was measured daily in the conventional system and weekly in the alternative system

In the conventional system, feed intake averaged 742 ± 239 g DM/bird/day for a total of 11,873 g DM/bird during the whole overfeeding period (91 to 107 days of age, Fig. 2). No variability was observed between animals as they all had the same meals (frequency and amount distributed). In the alternative system no refusals were observed during the Controlled Feeding (CF) period, and average pellet intakes were of 280 g/bird/day until 133 days of age and 180 g/bird/day from 133 to 161 days of age. Grass intake was not measured. In the alternative system, although grazing during the CF period was intended to increase crop volume, no difference was observed when comparing C_conv_ and C_alt_ birds (Table 1). During the AF period (161 to 245 days of age) feed intake averaged 268 g DM/bird/day (Fig. 2). Total feed intake over the 12 weeks of AF was of 23,050 g DM/bird (almost 2 fold the consumption in the conventional system during the 16 days of overfeeding).

**Table 1:** Growth and slaughter performances and chemical composition of the livers according to the experimental group

	Conventional		Alternative			*P*
	C_conv_ (*n* = 12)	OF (*n* = 16)	C_alt_ (*n* = 11)	NegSF (*n* = 9)	PosSF (*n* = 10)	
Growth and slaughter performances						
BW (g)	5279 ± 462^a^	8014 ± 424^b^	5589 ± 543^a^	6267 ± 502^c^	8835 ± 858^d^	***
Liver (g)	149 ± 22^a^	990 ± 213^b^	126 ± 17^c^	98 ± 10^d^	269 ± 56^e^	***
*%BW*	*2.8 ± 0.3*^*a*^	*12.4 ± 2.9*^*b*^	*2.3 ± 0.4*^*c*^	*1.6 ± 0.1*^*d*^	*3.0 ± 0.4*^*a*^	***
Abdominal fat (g)	112 ± 39^a^	ND	76 ± 25^b^	236 ± 51^c^	630 ± 108^d^	***
*%BW*	*2.1 ± 0.8*^*a*^	*ND*	*1.4 ± 0.5*^*b*^	*3.8 ± 0.7*^*c*^	*7.1 ± 1.0*^*d*^	***
Crop volume (mL)	306 ± 65	ND	264 ± 54	279 ± 70	249 ± 66	NS
Chemical composition of the liver						
DM (%)	31.0 ± 1.3^a^	64.8 ± 3.5^b^	31.7 ± 1.4^a^	31.0 ± 1.1^a^	46.5 ± 6.7^c^	***
MM (%)	1.1 ± 0.1^a^	0.4 ± 0.1^b^	1.1 ± 0.1^ac^	1.0 ± 0.1^c^	0.6 ± 0.2^d^	***
Lipids (%)	4.5 ± 0.8^a^	53.9 ± 4.9^b^	4.6 ± 0.5^a^	5.3 ± 0.6^c^	26.8 ± 9.5^d^	***
Total nitrogen (%)	16.1 ± 1.9^a^	8.0 ± 1.6^b^	15.4 ± 1.5^a^	18.1 ± 2.1^c^	12.6 ± 2.0^d^	***
Total glucose (μmol/g)	633 ± 134^a^	86 ± 27^b^	607 ± 116^ac^	459 ± 125^cd^	380 ± 97^d^	***
*μmol/g fat free liver*	*605 ± 127*^*a*^	*40 ± 17*^*b*^	*579 ± 111*^*ac*^	*434 ± 117*^*cd*^	*285 ± 97*^*d*^	***
Free glucose (μmol/g)	70 ± 11^a^	34 ± 7^b^	47 ± 7^c^	84 ± 10^d^	51 ± 9^c^	***
*μmol/g fat free liver*	*67 ± 11*^*a*^	*16 ± 4*^*b*^	*45 ± 6*^*c*^	*80 ± 10*^*d*^	*38 ± 10*^*c*^	***
Glycogen (μmol/g)	563 ± 135^a^	51 ± 27^b^	560 ± 113^a^	375 ± 130^c^	328 ± 93^c^	***
*μmol/g fat free liver*	*538 ± 128*^*a*^	*25 ± 16*^*b*^	*534 ± 108*^*a*^	*355 ± 123*^*c*^	*247 ± 89*^*c*^	***
Lactate (μmol/g)	6.9 ± 1.5^a^	9.6 ± 2.0^bc^	6.7 ± 0.8^a^	10.5 ± 0.8^b^	8.0 ± 1.5^ac^	***
*μmol/g fat free liver*	*6.6 ± 1.5*^*a*^	*4.4 ± .01*^*b*^	*6.4 ± 0.8*^*a*^	*10.0 ± 0.7*^*c*^	*5.8 ± 1.4*^*a*^	***

Values are presented as Mean ± SD

C_conv_ Control group before overfeeding, OF Overfed group, C_alt_ Control group before Ad libitum corn mixture feeding, NegSF Negative response group to spontaneous fattening induction, PosSF Positive response group to spontaneous fattening induction, BW Body Weight, DM Dry Matter, MM Mineral Matter, ND Not Determined

*** *P <* 0.001, *NS* Not Significant

Means within a row with different superscripts differ significantly (*P <* 0.05)

As expected, in the conventional system, OF birds (107 days of age) had a higher BW (+ 52%, *P* < 0.001) with an increased liver proportion (+ 9.6 pts., *P <* 0.001) compared to controls before overfeeding (C_conv_, 91 days of age) (Table 1). Similarly, in the alternative system, SF birds (245 days of age), regardless of response to steatosis induction, had a higher BW (6267 ± 502 g for the NegSF group and 8835 ± 858 g for the PosSF group, *P* < 0.05) compared to controls before the AF period (C_alt_, 161 days of age, 5589 ± 517 g). The proportion of the liver was slightly decreased in the NegSF group and increased in the PosSF group compared to C_alt_ birds (respectively 1.6 ± 0.1 and 3.0 ± 0.4 vs 2.3 ± 0.4%, *P <* 0.05) while that of abdominal fat increased strongly in both SF groups (3.8 ± 0.7% in the NegSF group and 7.1 ± 1% in the PosSF group vs 1.3 ± 0.5%, *P* < 0.001). After steatosis induction, liver proportion (− 10.8 pts. for the NegSF group and − 9.4 pts. for the PosSF group, *P* < 0.001) was significantly lower in the alternative system compared to the conventional one and body weight was reduced in the NegSF group (− 21%, *P <* 0.001) while it was increased in the PosSF group (+ 10%, *P* < 0.05) compared to the OF group. In the alternative system, BW (+ 41%, *P* < 0.001), liver proportion (+ 1.4 pts., *P* < 0.001) and abdominal fat proportion (+ 3.3 pts., *P* < 0.001) were, as expected, higher in the PosSF group compared to the NegSF group (Table 1 and Supplemental Fig. 2A-B).

### Liver biochemical results

As expected, in the conventional system, the higher liver weight observed in OF birds compared to controls (C_conv_) was associated with an increase in liver lipid content (+ 49 pts., *P* < 0.001) and lactate level (+ 39%, *P* < 0.01) and a decrease in glycogen (− 91%, *P <* 0.001), free glucose (− 51%, *P <* 0.001) and nitrogen (− 8 pts., *P <* 0.001) levels (Table 1).

In the alternative system, liver lipid content was higher in both SF groups compared to controls (C_alt_) and this difference was greater in the PosSF group (+ 22.2 pts., *P* < 0.001 vs + 0.7, *P* < 0.05 for the NegSF group). Conversely, glycogen levels were lower in both SF groups compared to controls (C_alt_) (− 33%, *P <* 0.05 for the NegSF group and − 42%, *P* < 0.001 for the PosSF group) with no significant difference between NegSF and PosSF groups. Free glucose and lactate levels were increased in the NegSF group compared to controls (C_alt_) (+ 79%, *P* < 0.001 and + 57%, *P <* 0.001 respectively) while no significant difference was observed between the PosSF and C_alt_ groups. Compared to controls (C_alt_), nitrogen levels were increased in the NegSF group (+ 2.7 pts, *P* < 0.01) and decreased in the PosSF group (− 2.8 pts., *P <* 0.01).

When comparing both systems after steatosis induction, lipid levels (*P <* 0.001) were lower in the alternative system compared to the conventional one while free glucose (*P <* 0.001), glycogen (*P <* 0.001) and nitrogen (*P <* 0.001) levels were higher, regardless of the response to SF. Only lactate levels were unaffected by the breeding system, although when expressed in proportion of the fat free liver weight lactate levels were increased in the alternative system compared to the conventional one regardless of the response to SF (*P* < 0.05).

### Ileal bacterial community

**Fig. 3:**
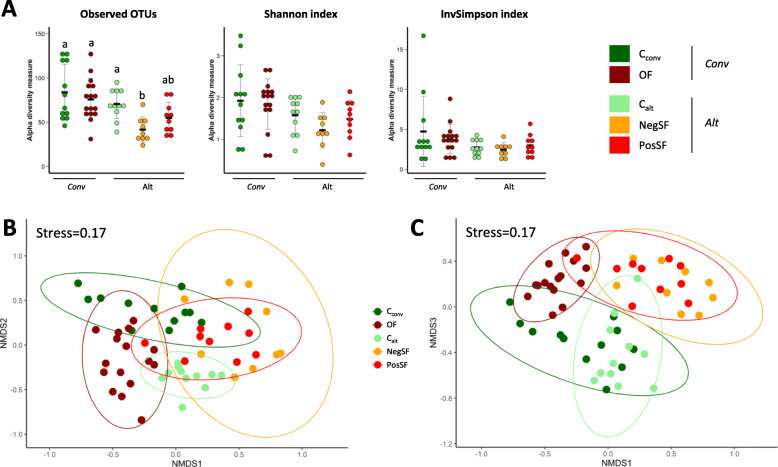
Alpha and Beta diversity of the microbial community according to the experimental group. **a** Alpha diversity, means are presented by black horizontal lines and SD as grey error bars; **b**-**c** nMDS representation of the beta diversity (Bray Curtis, stress = 0.17) according to (**b**) axes 1 and 2 and (**c**) axes 1 and 3. Conv: Conventional breeding system, Alt: Alternative breeding system. C_conv_: Control group before overfeeding, OF: Overfed group, C_alt_: Control group before Ad libitum corn mixture feeding, NegSF: Negative response groupe to spontaneous fattening induction, PosSF: Positive response groupe to spontaneous fattening induction. Within a plot, groups with different superscripts differ (*P <* 0.05) according to a Wilcoxon non parametric test

**Fig. 4:**
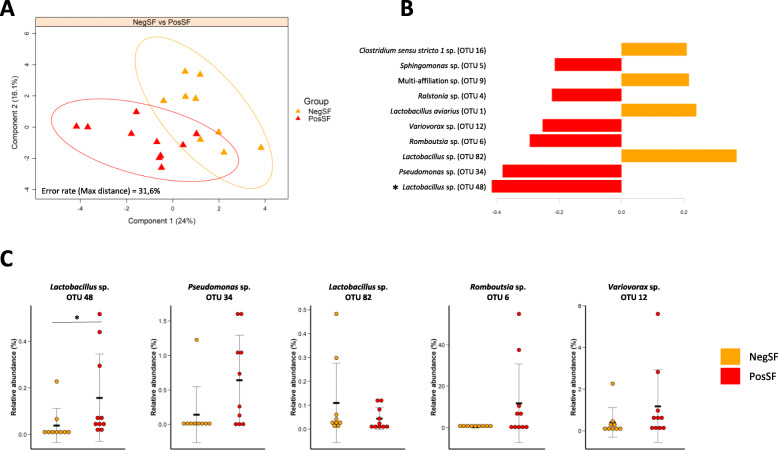
PLS-DA analysis on PosSF and NegSF groups based on OTUs. Analysis was performed using only OTUs with abundances > 0.1% in at least one group. **a** In the sample scatter plot each triangle represents a goose. Geese can be discriminated according to steatosis group on component 1. **b** Contribution level of the top 10 OTUs on component 1 are represented. The bar length represents the importance of the variable in the multivariate model. Bars are colored according to the experimental group with the highest mean abundance. **c** Relative abundance of the 5 most discriminant OTUs. NegSF: Negative response groupe to spontaneous fattening induction, PosSF: Positive response groupe to spontaneous fattening induction. * Significant difference between the PosSF and NegSF groups (*P* < 0.05) according to a Kruskal-Wallis non parametric test

Steatosis induction did not alter alpha diversity in the conventional system. In the alternative system the observed number of OTUs was altered in the NegSF group compared to C_alt_ birds (*P <* 0.05). All other diversity indices were unaffected (Fig. 3a). When comparing both systems, the observed number of OTUs was reduced in the NegSF group compared to the OF group (*P <* 0.05). Regarding beta-diversity all experimental groups could clearly be distinguished using the Bray-Curtis dissimilarity method and Adonis pairwise tests, except the two SF groups that were similar (Fig. 3b-c, Supplemental Table 1). In both systems, steatosis induction (OF and SF) resulted in variations in beta-diversity compared to their respective controls (C_conv_ with R2-Adonis of 0.23, *P* < 0.01 and C_alt_ R2-Adonis ranging between 0.29 and 0.3, *P <* 0.01). When comparing steatosis induction modes OF birds differed from NegSF (R2-Adonis of 0.25, *P <* 0.01) and PosSF (R2-Adonis of 0.21, *P <* 0.01) birds. No differences in beta-diversity were observed between birds with different aptitudes to spontaneous steatosis (NegSF vs PosSF). In order to evaluate whether some bacterial taxa could distinguish these two groups a PLS-DA analysis was performed keeping OTUs with abundances above 0.1% in at least one of the two groups (Fig. 4a-b). A separation was obtained and the top 10 most contributing OTUs were identified. Among those, OTU48 (*Lactobacillus* sp.) was differentially represented in PosSF and NegSF groups in univariate analyses (0.2 vs 0% in the NegSF group, *P* = 0.01) (Fig. 4c, Supplemental Table 2).

**Fig. 5:**
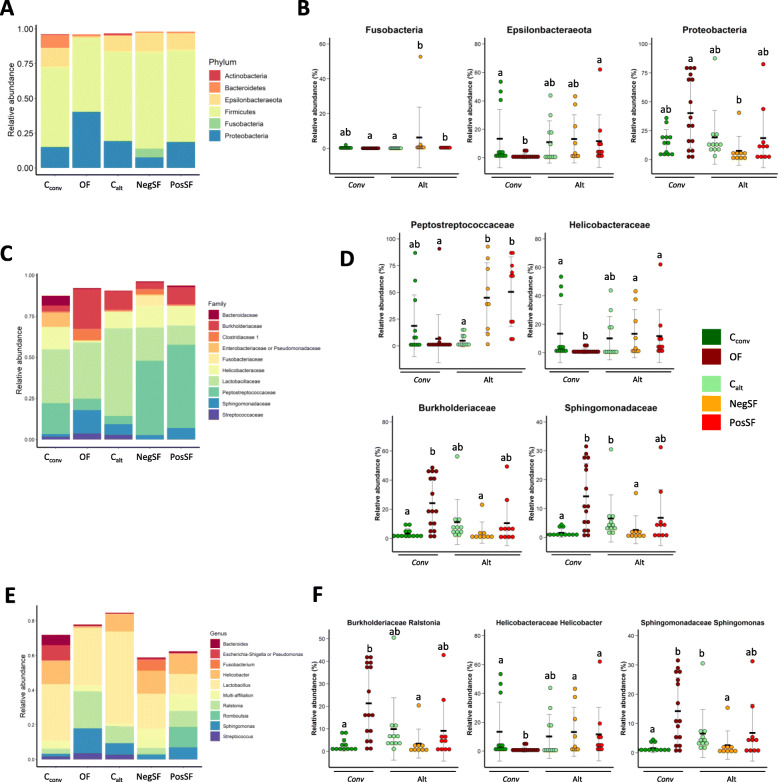
Microbial composition according to the experimental groups at (**a-b**) Phylum, (**c**-**d**) Family and (**e**-**f**) Genus level. In panels (**c**) and (**e**) only the 10 most abundant Families (**c**) and Genera (**e**) are depicted. Means are presented by black horizontal lines and SD as grey error bars. Conv: Conventional breeding system, Alt: Alternative breeding system. C_conv_: Control group before overfeeding, OF: Overfed group, C_alt_: Control group before Ad libitum corn mixture feeding, NegSF: Negative response groupe to spontaneous fattening induction, PosSF: Positive response groupe to spontaneous fattening induction. Within a plot, groups with different superscripts differ (*P* < 0.05) according to a Wilcoxon non parametric test

When performing taxonomic affiliations on OTUs with abundances above 0.1% in at least one experimental group 6 phyla, 31 families and 52 genera were observed in the overall dataset. Results are presented at phylum (Fig. 5a-b; Supplemental Table 3), family (Fig. 5c-d; Supplemental Table 4) and genus levels (Fig. 5e-f; Supplemental Table 5). *Firmicutes* and *Proteobacteria* were the most abundant phyla with *Lactobacillaceae* and *Peptostreptococcaceae* of the first mentioned phylum being the most abundant families.

In the conventional system, the proportions of *Epsilonbacteraeota* (− 12.3 pts., *P* < 0.05) and its sole genus *Helicobacter* from the dominant family *Helicobacteraceae* were lower after steatosis induction (OF) compared to C_conv_ birds while the proportion of *Proteobacteria* was not significantly modulated but the proportions of two of its dominant families, *Burkholderiaceae* and *Sphingomonadaceae*, were significantly increased (+ 20.8 pts., *P* < 0.01 and + 12.7 pts., *P <* 0.01 respectively). These increases were attributable to that of the main genera of aforementioned families, *Ralstonia* (+ 18.5 pts., *P* < 0.01) and *Sphingomonas* (+ 12.7 pts., *P <* 0.01) respectively.

In the alternative system, the proportion of *Fusobacteria* was higher after steatosis induction compared to C_alt_ birds regardless of the steatosis level (+ 6.3 pts., *P <* 0.01 in the NegSF group and + 0.2 pts., *P* < 0.001 in the PosSF group) reflecting the levels of its sole representative genus *Fusobacterium* of the *Fusobacteriaceae* family. The proportion of *Firmicutes* was unaffected, but that of its two main families was modulated with the proportion of *Peptostreptococcaceae* being drastically increased after steatosis induction compared to C_alt_ birds regardless of steatosis level (+ 40.2 pts., *P* < 0.01 for the NegSF group and + 45.6 pts., *P <* 0.01 for the PosSF group) while the level of *Lactobacillaceae* decreased solely in the PosSF group (− 41.6 pts., *P <* 0.01) reflecting the decrease in its main genus *Lactobacillus* (− 41.5 pts., *P <* 0.01). Although the proportion of *Proteobacteria* was unaffected by steatosis induction, that of one of its main families, *Sphingomonadaceae*, decreased solely in the NegSF group as a result of the decrease of its sole genus *Sphingomonas* (− 4.0 pts., *P <* 0.05).

Regardless of the taxonomic rank considered (phylum, family and genus) no significant differences were observed between animals differing in aptitudes to spontaneous steatosis (NegSF vs PosSF).

When comparing systems after steatosis induction, SF birds had consistently higher levels of *Fusobacteria* compared to the OF birds (+ 6.3 pts., *P* < 0.01 for the NegSF group and + 0.2 pts., *P* < 0.001 for the PosSF group). The level of *Proteobacteria* was lower only in the NegSF group (− 32.8 pts., *P* < 0.05) as a result of the decrease in proportion of its two main families, *Burkholderiaceae* and *Sphingomonadaceae* (− 20.3 pts., *P* < 0.01 and − 11.7 pts., *P* < .0.01 respectively), the PosSF group having intermediate values. These decreases were attributable to the decreases in the main genera of the aforementioned families, *Ralstonia* (− 17.9 pts., *P <* 0.01) and *Sphingomonas* (− 11.7 pts., *P <* 0.01) respectively. The level of *Epsilonbacteraeota* was higher only in the PosSF group (+ 10.6 pts., *P <* 0.05) although the level of the sole representative genus, *Helicobacter*, of its main family *Helicobacteraceae* was greater in both SF groups (+ 12.3 pts., *P <* 0.05 for the NegSF group and + 10.7 pts., *P <* 0.05 for the PosSF group). *Firmicutes* were unaffected by breeding system, although the level of one of its main families, *Peptostreptococcaceae*, was consistently higher compared to the OF birds (+ 38.4 pts., *P <* 0.01 for the NegSF group and + 43.8 pts., *P <* 0.01 for the PosSF group).

## Discussion

Conventional overfeeding in waterfowl is today questioned for animal welfare reasons. Although spontaneous steatosis has been experimentally induced in greylag geese, great variability has been observed in both feed intake and steatosis level [6, 9]. Feed intake is the main contributor to steatosis, but other factors such as the digestive microbiota could also be involved. The present study thus aimed to address two issues: (i) evaluate whether microbial composition differed with steatosis-inducing mode; (ii) elucidate whether a digestive microbial signature could be associated with different aptitudes to spontaneous liver steatosis.

Biochemical composition of the livers and microbiota differed considerably in response to the steatosis induction systems, the conventional one, based on overfeeding, and the alternative one, based on a spontaneous ad libitum feeding, although birds were given the same diet during the steatosis induction period. The alternative steatosis inducing system led to an 84% lower liver weight than in the conventional steatosis inducing system, mainly due to a considerably lower daily feed intake (− 64%), although feed intake on the overall corn mixture feeding period was almost doubled in the alternative system as previously reported [8]. The increase in liver weight observed in birds with a positive response to spontaneous steatosis stimulation was partly linked to an increased lipogenesis, as observed to a greater extent in the overfed birds. Indeed, regardless of the type of steatosis induction, a decrease in glycogen concentration associated with an increased liver lipid content were observed as previously described in overfed ducks [33]. Furthermore, lipid storage in adipose tissues (appreciated through the variations in abdominal fat weight) strongly increased in birds developing a spontaneous steatosis (7.1% of BW), surpassing levels commonly observed with conventional overfeeding (4.7–5.7%) [7, 17, 34]. This could indicate a preferential storage of lipids in these sites before, when saturated, storing in the liver.

As previously described in domesticated geese [15, 35–37], and in birds in general [38], *Firmicutes* and *Proteobacteria* (> 70% of sequences in all groups) were the two main phyla in the Greylag goose (*Anser anser*) ileal microbiota. Microbial composition did however strongly differ according to the steatosis induction system.

In the conventional overfeeding system we described a drastic decrease in *Helicobacter* (− 12.4 Pts) counterbalanced by an increase in *Ralstonia* (+ 18.5 pts) and *Sphingomonas* (+ 12.7 pts) after overfeeding. Previous studies emphasized an increase in the abundance of *Lactobacillus* in the ileum of overfed ducks [12, 13]. In our study, we were unable to evidence such an increase and no difference was observed between steatosis inducing systems. This might be due to the higher abundance of the *Lactobacillus* genus in the control groups (in comparison to previous work). However, our results are consistent with those of Liu et al. [15] who showed that, in geese, overfeeding increased the abundance of *Lactobacillus* in the duodenum, jejunum and ceca but not in the ileum. *Lactobacillus* levels might be modulated by overfeeding in geese, but not in the same intestinal segments as in ducks. It would be of interest to evaluate the impact of steatosis induction mode on the microbial composition in the different segments of the digestive tract. In the alternative system, the main variations concerned the *Peptostreptococcaceae* family whose proportion was drastically increased after steatosis induction regardless of steatosis level (+ 40.2 to + 45.6 pts) while the level of *Lactobacillus* decreased solely in birds with a positive response to spontaneous steatosis stimulation (− 41.5 pts). These results are the first to examine the microbial response to spontaneous steatosis stimulation in greylag geese.

In the present study, the major effect of steatosis induction mode on the microbiota relies on the notably higher levels of *Romboutsia* (*Peptostreptococcaceae*) in birds with a positive response to spontaneous steatosis stimulation compared to overfed birds while levels of *Ralstonia*, *Variovorax* (*Burkholderiaceae*) and *Sphingomonas* (*Sphingomonadaceae*) were reduced only in the birds with a negative response to spontaneous steatosis stimulation compared to overfed birds; birds with a positive response to spontaneous steatosis stimulation having intermediate values. Previous studies in geese [15] and ducks [12, 13] did not highlight changes in the abundance of these genera with steatosis induced by overfeeding even though great differences relative to controls were observed in our study. In other species little data is available on the association between these genera and lipid metabolism, steatosis and obesity. Several studies evidence increases in *Romboutsia* levels with obesity related metabolic disorders in humans [39] and in mice fed high fat [40] or high starch [41] diets, although no causal link has been established between this genus and obesity related traits to date. At first glance our results are in contrast with previous ones as *Romboutsia* levels are higher in birds with a positive response to spontaneous steatosis stimulation compared to overfed ones, despite a lower liver weight. Great metabolic changes are however susceptible to occur in birds with a positive response to spontaneous steatosis stimulation, with, as previously mentioned, a higher abdominal fat proportion compared to levels classically observed with overfeeding (7.1% vs 4.7–5.7%) [7, 17, 34]. It would then be of interest to further evaluate these metabolic changes and their associations with the gut microbiota, notably the *Romboutsia* genus. Interestingly, a recent study evidenced that the fecal abundance of *Ralstonia picketti* was increased in obese humans with pre-diabetes or type 2 diabetes and that a causal association between this species and impaired glucose tolerance in dietary induced obese mice could be assessed [42]. However, in the present study, OTUs belonging to the *Ralstonia* genus were not affiliated to a sole distinct species. Another recent study also evidenced correlations between the plasmatic levels of *Variovorax* and *Sphingomonas* and liver fibrosis in obese patients [43]. These correlations were not observed when examining the fecal microbiota. It is also to be noted that the level of *Proteobacteria* – phylum to which *Ralstonia, Variovorax* and *Sphingomonas* belong – is consistently higher in steatosis and non-alcoholic steatohepatitis (NASH) patients [44] compared to controls. Although hepatic metabolism differs greatly between birds and mammals it would be of interest to further evaluate the causal link between *Ralstonia, Variovorax* and *Sphingomonas* and steatosis in waterfowl.

The discrepancies between steatosis stimulation systems could be explained by five factors inherent to the two systems: the age, the type of feed ingested prior to the steatosis induction period, the daily feed intake during steatosis induction, the duration of the steatosis induction period, the extent of the induced-steatosis and/or a combination of these factors. It is well documented that microbial composition changes with age in the early life in all species, including birds [45, 46]. In ducks the microbial composition stabilizes between 65 and 84 days of age [47]. Similar trends could be hypothesized in geese. In that context, its impact during the steatosis induction periods would be minimal. Comparing animals fed a pellet diet or fed by grazing on a pasture, Xu et al. [48] evidenced major changes in microbial composition at cecal level, with an increase in *Firmicutes* and a decrease in *Bacteroidetes* associated with an increased richness diversity index with grazing. However, Guo et al. [49] did not evidence any changes in cecal alpha-diversity or abundances at phylum level with increased grass inclusion in the diet. Furthermore, no data are available on the impact of prior grass ingestion when the diet is changed. Altogether, it is difficult to evaluate whether the access to a pasture prior spontaneous steatosis induction would affect microbial composition. Moreover, long term feeding with the same diet can potentially affect microbial diversity. Studies in goats showed that richness decreased with long term vs short term feeding of a high concentrate diet [50]. The duration of the corn mixture feeding could have affected ilea microbial composition in the geese. Finally, regarding the feed intake, studies in mammals and birds have shown that feed restriction can also modulate the intestinal microbiota [51–53]. Therefore, it is possible that an increase in feed intake might influence the microbial composition as well. Altogether, considering available data in the literature, it seems worthwhile to further study the evolution of the microbiota during the first days of induction in order to determine the effect of the amount of feed ingested and the duration of the steatosis induction upon the microbial composition.

In the alternative system, after 12 weeks of spontaneous corn mixture feeding, the birds with the smallest livers were also the ones with the least subcutaneous fat, demonstrating a reduced fattening of those animals at a global scale. The absence of increase in liver weight in the birds with a negative response to spontaneous steatosis stimulation, despite an apparent metabolic reorientation, may be due to (i) a lower feed intake of those animals as previously observed by Fernandez et al. [9] and/or (ii) a reduced feed efficiency, associated with differences in microbial composition as observed in pigs and chicken [54–56]. In mice, Le Roy et al. [57] evidenced a causal link between microbial composition and steatosis onset under high fat diet feeding after inoculation of the microbiota of donor mice having developed or not a steatosis themselves. Although no causal association has been described to date in birds, a correlation between microbiota and steatosis severity (Fatty liver hemorrhagic Syndrome) has been observed in chicken [58]. However, in our study when comparing geese differing in steatosis level after 12 weeks of spontaneous corn mixture feeding no microbial signature distinguishing them was evidenced. These results could indicate that the differences in aptitudes to spontaneous steatosis in geese are mainly dependent on feed intake and host intrinsic parameters such as genetics. However, one low abundant OTU affiliated to *Lactobacillus* sp., discriminating the two steatosis response groups in PLS-DA analysis, was more abundant in birds having developed a spontaneous steatosis compared to those that hadn’t. Interestingly, the *Lactobacillus* genus has been associated with steatosis in overfed ducks [12, 13]. It would be of interest to further evaluate the associations between steatosis and species belonging to the *Lactobacillus* genus.

Compared to previous studies on spontaneous steatosis induction after 12 to 13 weeks of corn feeding, liver weights were overall lower in our experiment (158 g vs > 440 g) [6, 9, 17]. On the overall corn feeding period, intake could not account for this difference, as it was only slightly reduced compared to the study of Guy et al. [6] (268 vs 277 g DM/bird/day; − 3%). Fernandez et al. [9] showed a high correlation between feed intake over the first three weeks of spontaneous corn feeding and liver weight after 12 weeks of spontaneous corn feeding. Over these three weeks, feed intake was lower in our study (246 g DM/bird/day) compared to the study of Guy et al. [6] (301 g DM/bird/day; − 18%) thus partly explaining the differences in liver weights. Unlike previous studies, birds had access to a grassland before corn feeding that could have impacted on the subsequent fattening. Vitamins C and E for example modulate lipid metabolism in mammals [59, 60]. However, neither the nature (plant types) and composition of the grass nor intake level were measured in this study. It is therefore difficult to evaluate the nutritional impact of this procedure upon the subsequent steatosis observed.

## Conclusion

Correlations between microbiota and steatosis are being extensively studied in mammals, and causal relations have been evidenced. Our study is the first to evaluate the intestinal microbial composition in association with steatosis, whether spontaneous or induced by overfeeding, in geese. We evidenced that performances, biochemical composition of the livers and microbiota differed considerably in response to steatosis stimulation mode. We namely identified the genus *Romboutsia* to be overrepresented in birds developing a spontaneous steatosis in comparison to those submitted to conventional overfeeding while the genera *Ralstonia*, *Variovorax* and *Sphingomonas* were underrepresented only in birds that did not develop a spontaneous steatosis compared to conventionally overfed ones, birds developing a spontaneous steatosis having intermediate values. Secondly, no overall differences in microbial composition were evidenced in association with different aptitudes to spontaneous steatosis. Thus, unlike what can be observed in mammals, no clear microbial signature associated with spontaneous steatosis level was identified. It would however be of interest to further investigate the correlations and potential causal associations between the digestive microbiota and steatosis in waterfowl in studies with greater disparities in steatosis level. Furthermore, migratory birds have a specific ability to develop a transitory steatosis during the pre-migratory period. However, the biomimetic features of the alternative system presented here are limited to certain features such as the light stimulation and the period of the year. Animals being reared with a specific high starch diet over a prolonged timespan it would be of interest to evaluate the associations between microbiota and metabolic and immune mechanisms occurring in the liver and adipose tissue under spontaneous steatosis as initiated in control [61] and overfed geese [15].

## Supplementary Information

**Additional file 1: Supplemental Figure 1**. Ambiant temperature and hygrometry in the experimental facility during the Ad libitum feeding (AF) period in the alternative breeding system (161 to 245 days of age). **Supplemental Figure 2**. Selection of PosSF and NegSF birds after the Ad libitum feeding period (245 days of age) in the alternative system. A) Body composition and B) biochemical composition of the livers in the different groups.

**Additional file 2: Supplemental Table 1.** Pairwise Adonis tests between groups. **Supplemental Table 2** Relative abundance (%) of the top 10 OTUs contributing to PLS-DA axis 1 on PosSF and NegSF groups. **Supplemental Table 3**. Relative taxonomic abundance (in %) in ileal content at phyla level according to the experimental group. **Supplemental Table 4**. Relative taxonomic abundance (in %) in ileal content at family level according to the experimental group. **Supplemental Table 5**. Relative taxonomic abundance (in %) in ileal content at genus level according to the experimental group.

**Additional file 3.** Biochemical and slaughter performances data set.

## Data Availability

All raw sequences were deposited in the NCBI Sequence Read Archive (SRA) under accession number PRJNA634833. Data concerning slaughter performances and biochemical composition of the livers are available as supplemental material to this article.

## Abbreviations

AF: Ad libitum feeding
Alt.: Alternative rearing system
AMEn: Apparent Metabolisable Energy, nitrogen-corrected
BW: Body Weight
C_alt_: Control group before ad libitum corn mixture feeding
C_conv_: Control group before overfeeding
CF: Controlled Feeding
CO: Conventional Overfeeding
Conv: Conventional rearing system
CP: Crude Protein
DM: Dry Matter
MM: Mineral Matter
NAFLD: Non Alcoholic Fatty Liver Diseases
NASH: Non-Alcoholic Steatohepatitis
NegSF: Negative response to spontaneous steatosis stimulation group
nMDS: Non-Metric Dimensional Scaling
OF: Overfed group
OTU: Operational Taxonomic Unit
PLS-DA: Partial Least Squares Discriminant Analyzis
PosSF: Positive response to spontaneous steatosis stimulation group
SF: Spontaneous Fattening
