# Supplementary material for: The intestinal microbial composition in Greylag geese differs with steatosis induction mode: spontaneous or induced by overfeeding
Source: Anim Microbiome. 2021 Jan 6;3:6. doi: 10.1186/s42523-020-00067-z (PMC7934468; doi:10.1186/s42523-020-00067-z)
Supplement: Supplementary file 1 — Additional file 1: Supplemental Figure 1. Ambiant temperature and hygrometry in the experimental facility during the Ad libitum feeding (AF) period in the alternative breeding system (161 to 245 days of age). Supplemental Figure 2. Selection of PosSF and NegSF birds after the Ad libitum feeding period (245 days of age) in the alternative system. A) Body composition and B) biochemical composition of the livers in the different groups. [file 42523_2020_67_MOESM1_ESM.pdf]

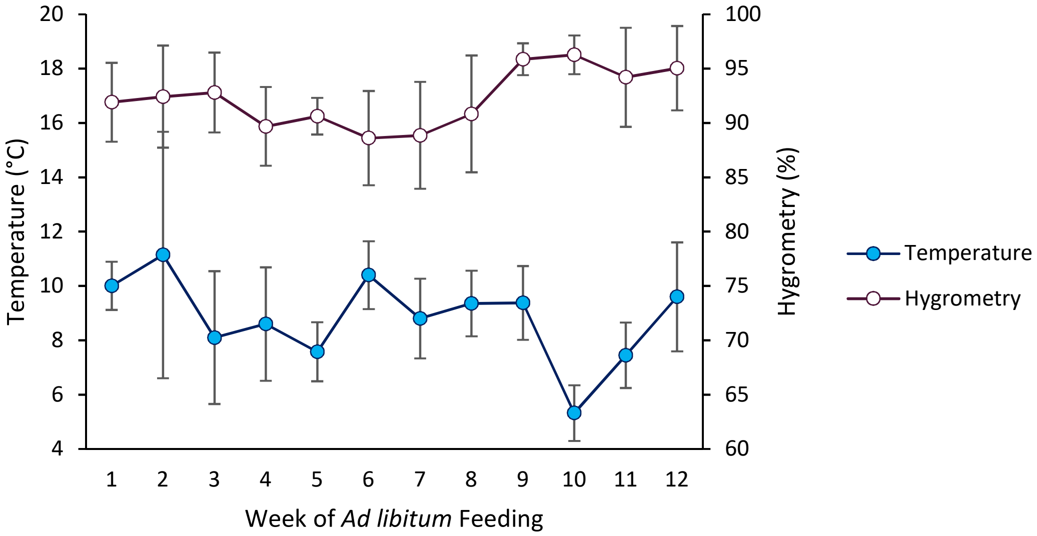

Supplemental Figure 1. Ambiant temperature and hygrometry in the experimental facility during the *Ad libitum* feeding (AF) period in the alternative breeding system (161 to 245 days of age)

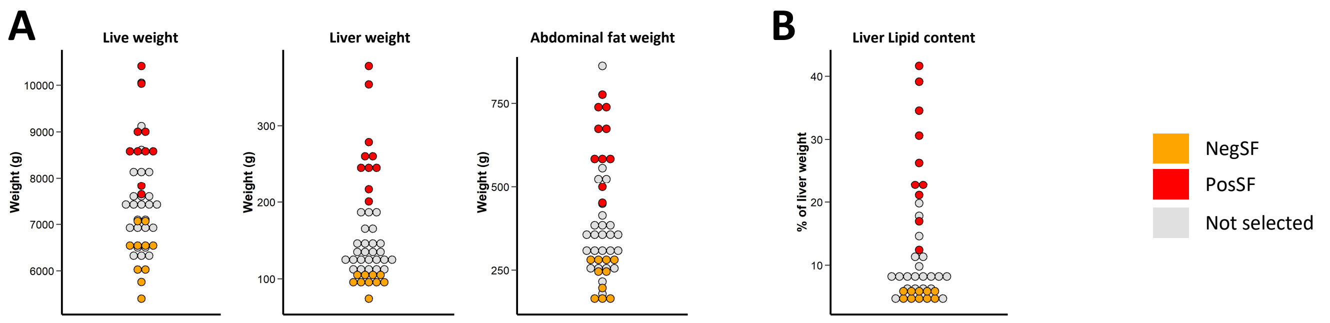

Supplemental Figure 2. Selection of PosSF and NegSF birds after the *Ad libitum* feeding period (245 days of age) in the alternative system. A) Body composition and B) biochemical composition of the livers in the different groups.
