# Supplementary material for: The intestinal microbial composition in Greylag geese differs with steatosis induction mode: spontaneous or induced by overfeeding
Source: Anim Microbiome. 2021 Jan 6;3:6. doi: 10.1186/s42523-020-00067-z (PMC7934468; doi:10.1186/s42523-020-00067-z)
Supplement: Supplementary file 2 — Additional file 2: Supplemental Table 1. Pairwise Adonis tests between groups. Supplemental Table 2 Relative abundance (%) of the top 10 OTUs contributing to PLS-DA axis 1 on PosSF and NegSF groups. Supplemental Table 3. Relative taxonomic abundance (in %) in ileal content at phyla level according to the experimental group. Supplemental Table 4. Relative taxonomic abundance (in %) in ileal content at family level according to the experimental group. Supplemental Table 5. Relative taxonomic abundance (in %) in ileal content at genus level according to the experimental group. [file 42523_2020_67_MOESM2_ESM.pdf]

Supplemental table 1. Pairwise Adonis tests between groups

R2		Conventional		Alternative		
		C_{conv}	OF	C_{alt}	NegSF	PosSF
Conv.	C_{conv}					
	OF	0.235**				
Alt.	C_{alt}	0.140**	0.240**			
	NegSF	0.128*	0.254**	0.288**		
	PosSF	0.167**	0.212**	0.301**	NS	

R2 Adonis	<0.20	0.20-0.25	0.25-0.30	>0.30

High R2-Adonis indicates well separated groups.
C_{conv}: Control group before overfeeding, OF: Overfed group, C_{alt}: Control group before *Ad libitum* corn mixture feeding, NegSF: Negative response group to spontaneous fattening induction, PosSF: Positive response group to spontaneous fattening induction.
NS: Not Significant.
* p-adjusted values (« false discovery rate » test) * P<0.05, ** P<0.01.

Supplemental table 2 Relative abundance (%) of the top 10 OTUs contributing to PLS-DA axis 1 on PosSF and NegSF groups

	Taxonomic affiliation				Group		*P*
	Phylum	Family	Genus	Species	NegSF (n = 9)	PosSF (n = 10)	
OTU 48	*Firmicutes*	*Lactobacillaceae*	*Lactobacillus*	Multi-affiliation	0.0±0.1	0.2±0.2	*
OTU 34	*Proteobacteria*	*Pseudomonadaceae*	*Pseudomonas*	Multi-affiliation	0.1±0.4	0.6±0.6	NS
OTU 82	*Firmicutes*	*Lactobacillaceae*	*Lactobacillus*	Multi-affiliation	0.1±0.2	0.0±0.0	NS
OTU 6	*Firmicutes*	*Peptostreptococcaceae*	*Romboutsia*	unknown species	0.5±0.7	11.8±18.9	NS
OTU 12	*Proteobacteria*	*Burkholderiaceae*	*Variovorax*	Multi-affiliation	0.4±0.7	1.2±1.7	NS
OTU 1	*Firmicutes*	*Lactobacillaceae*	*Lactobacillus*	*Lactobacillus aviarius*	19.9±22	11.3±12.2	NS
OTU 4	*Proteobacteria*	*Burkholderiaceae*	*Ralstonia*	Multi-affiliation	3.5±6.5	9.2±13.5	NS
OTU 9	*Firmicutes*	*Clostridiaceae 1*	Multi-affiliation	Multi-affiliation	0.1±0.1	0.1±0.4	NS
OTU 5	*Proteobacteria*	*Sphingomonadaceae*	*Sphingomonas*	Multi-affiliation	2.6±4.8	6.8±9.7	NS
OTU 16	*Firmicutes*	*Clostridiaceae 1*	*Clostridium sensu stricto 1*	Multi-affiliation	2.6±7.6	0.1±0.1	NS

Values are presented as Mean±SD.

NegSF: Negative response group to spontaneous fattening induction, PosSF: Positive response group to spontaneous fattening induction

Multi-affiliation: In FROGS, when performing taxonomic affiliation, if several blastn results have identical scores for an OTU, a taxonomy is determined for each hit at each taxonomic level. If these taxonomies differ across hits, the first level of conflict and all lower ones are set to ‘Multi-affiliation.

* P<0.05, NS: Not Significant.

Supplemental table 3. Relative taxonomic abundance (in %) in ileal content at phyla level according to the experimental group.

Relative Abundance (%)	Conventional		Alternative			*P*	*P adjust*[1]
	C_{conv} (n = 12)	OF (n = 16)	C_{alt} (n = 11)	NegSF (n = 9)	PosSF (n = 10)		
Actinobacteria	0.6±0.7^{a}	0.1±0.1^{b}	1.2±1.2^{a}	0.0±0.0^{c}	0.0±0.0^{c}	***	***
Bacteroidetes	9.3±17.5^{a}	1.4±1.4^{a}	0.6±0.4^{a}	0.8±1.4^{a}	1.0±1.7^{a}	*	*
Epsilonbacteraeota	13.4±20.4^{a}	1.1±1.7^{b}	11.1±14.8^{ab}	13.3±16.9^{ab}	11.7±18.5^{a}	*	*
Firmicutes	57.7±22.8	53.1±33.3	64.7±24.3	70.0±21.8	66.4±27.7	NS	NS
Fusobacteria	0.3±0.6^{ab}	0.0±0.0^{a}	0.0±0.0^{a}	6.3±17.4^{b}	0.2±0.2^{b}	***	***
Proteobacteria	14.9±11.0^{ab}	40.4±30.4^{a}	19.3±23.3^{ab}	7.6±12.6^{b}	18.6±25.6^{ab}	**	*

Values are presented as Mean±SD.

C_{conv}: Control group before overfeeding, OF: Overfed group, C_{alt}: Control group before *Ad libitum* corn mixture feeding, NegSF: Negative response group to spontaneous fattening induction, PosSF: Positive response group to spontaneous fattening induction.

* P<0.05, ** P<0.01, *** P<0.001, NS: Not Significant.

Means within a row with different superscripts differ significantly (p<0.05).

[1]P adjusted values according to the « false discory rate » (fdr) test.

Supplemental table 4. Relative taxonomic abundance (in %) in ileal content at family level according to the experimental group.

Relative Abundance (%)	Conventional		Alternative				
	C_{conv} (n = 12)	OF (n = 16)	C_{alt} (n = 11)	NegSF (n = 9)	PosSF (n = 10)	*P*	*P adjust*[1]
Actinobacteria							
Microbacteriaceae	0.0±0.0^{a}	0.0±0.0^{b}	0.5±0.6^{c}	0.0±0.0^{ab}	0.0±0.0^{ab}	***	***
Micrococcaceae	0.6±0.7^{a}	0.1±0.1^{b}	0.7±0.7^{a}	0.0±0.0^{c}	0.0±0.0^{bc}	***	***
Bacteroidetes							
Bacteroidaceae	6.0±13.0	0.6±0.7	0.3±0.4	0.6±1.4	0.9±1.7	NS	NS
Rikenellaceae	1.6±2.9^{a}	0.4±0.4^{a}	0.1±0.1^{b}	0.1±0.1^{b}	0.1±0.1^{b}	***	***
Tannerellaceae	0.8±1.9	0.0±0.0	0.0±0.0	0.0±0.0	0.0±0.0	*	NS
Epsilonbacteraeota							
Campylobacteraceae	0.0±0.0^{ab}	0.1±0.1^{a}	0.9±2.9^{ab}	0.0±0.0^{ab}	0.0±0.0^{b}	*	*
Helicobacteraceae	13.4±20.4^{a}	1.0±1.7^{b}	10.2±15.2^{ab}	13.3±16.9^{a}	11.7±18.5^{a}	**	*
Firmicutes							
Clostridiaceae 1	0.7±0.9	6.9±16.2	0.9±0.5	3.2±7.5	0.7±0.7	NS	NS
Clostridiales vadinBB60 group	0.5±0.9^{a}	0.1±0.1^{ab}	0.1±0.1^{b}	0.1±0.2^{b}	0.0±0.0^{b}	***	**
Enterococcaceae	0.8±0.9^{a}	0.2±0.3^{ab}	0.6±1.1^{ab}	0.0±0.1^{b}	1.1±3.1^{ab}	**	**
Erysipelotrichaceae	0.0±0.0^{a}	0.1±0.2^{a}	1.7±1.9^{b}	0.9±1.4^{b}	1.8±2.9^{b}	***	***
Lachnospiraceae	0.5±0.4^{a}	0.6±0.9^{ab}	0.1±0.2^{ab}	0.1±0.1^{b}	0.1±0.1^{ab}	**	**
Lactobacillaceae	32.7±24.6^{ab}	34.3±35.6^{ab}	53.3±24.9^{a}	20.2±21.9^{ab}	11.7±12.3^{b}	*	*
Peptostreptococcaceae	18.9±28.9^{ab}	6.8±22.5^{a}	5.0±5.9^{a}	45.2±32.4^{b}	50.6±32.4^{b}	***	***
Ruminococcaceae	2.0±3.9^{a}	0.6±0.7^{a}	0.2±0.2^{a}	0.2±0.2^{a}	0.1±0.1^{a}	**	*
Streptococcaceae	1.7±2.2^{a}	3.6±11.4^{a}	2.8±4.3^{a}	0.1±0.1^{b}	0.1±0.3^{b}	***	***
Fusobacteria							
Fusobacteriaceae	0.3±0.6^{ab}	0.0±0.0^{a}	0.0±0.0^{a}	6.3±17.4^{b}	0.2±0.2^{b}	***	***
Proteobacteria							
Burkholderiaceae	3.5±3.1^{a}	24.3±18.0^{b}	11.3±15.4^{ab}	4.0±7.3^{a}	10.6±15.6^{ab}	**	**
Desulfovibrionaceae	0.4±0.4^{ab}	1.2±1.6^{a}	0.3±0.2^{ab}	0.1±0.2^{b}	0.2±0.2^{ab}	**	**

Enterobacteriaceae or *Pseudomonadaceae*	8.7±9.5^{a}	0.4±0.6^{b}	0.3±0.5^{b}	0.7±1.3^{b}	0.3±0.4^{b}	***	***
Pseudomonadaceae	0.0±0.0^{a}	0.0±0.0^{a}	0.0±0.0^{a}	0.1±0.4^{a}	0.6±0.7^{a}	*	*
Rhizobiaceae	0.0±0.0^{a}	0.0±0.0^{b}	0.5±0.4^{c}	0.0±0.0^{ab}	0.0±0.0^{a}	***	***
Sphingomonadaceae	1.6±1.5^{a}	14.3±11.4^{b}	6.6±8.2^{b}	2.6±4.8^{a}	6.8±9.7^{ab}	***	**

Values are presented as Mean±SD.

Families are sorted according to Phylum. Only families with abundances above 0.5% in at least one experimental group are presented in the table.

C_{conv}: Control group before overfeeding, OF: Overfed group, C_{alt}: Control group before *Ad libitum* corn mixture feeding, NegSF: Negative response group to spontaneous fattening induction, PosSF: Positive response group to spontaneous fattening induction

* P<0.05, ** P<0.01, *** P<0.001, NS: Not Significant.

Means within a row with different superscripts differ significantly (p<0.05).

[1]P adjusted values according to the « false discory rate » (fdr) test.

Supplemental table 5. Relative taxonomic abundance (in %) in ileal content at genus level according to the experimental group.

	Conventional		Alternative				
	Control (n = 12)	OF (n = 16)	FRP (n = 11)	NegSFS (n = 9)	PosSFS (n = 10)	*P*	*P adjust*
Bacteroidaceae							
Bacteroides	6.0±13.0	0.6±0.7	0.3±0.4	0.6±1.4	0.9±1.7	NS	NS
Burkholderiaceae							
Ralstonia	2.9±2.8^{a}	21.4±15.9^{b}	10.0±13.9^{ab}	3.5±6.5^{a}	9.2±13.5^{ab}	***	**
Variovorax	0.4±0.4^{a}	2.4±1.8^{b}	1.1±1.4^{ab}	0.4±0.7^{a}	1.2±1.7^{ab}	**	**
Campylobacteraceae							
Campylobacter	0.0±0.0^{ab}	0.1±0.1^{a}	0.9±2.9^{ab}	0.0±0.0^{ab}	0.0±0.0^{b}	*	*
Clostridiaceae 1							
Candidatus Arthromitus	0.6±0.9	1.0±0.8	0.9±0.5	0.6±0.8	0.5±0.5	NS	NS
Clostridium sensu stricto 1	0.1±0.1^{a}	0.0±0.1^{bc}	0.0±0.0^{b}	2.6±7.6^{a}	0.1±0.1^{ac}	***	**
Multi-affiliation	0.0±0.0	5.9±16.2	0.0±0.0	0.1±0.1	0.1±0.4	*	NS
Desulfovibrionaceae							
Bilophila	0.1±0.1^{a}	0.5±0.9^{a}	0.3±0.2^{a}	0.1±0.2^{a}	0.1±0.1^{a}	**	*
Desulfovibrio	0.1±0.2^{ab}	0.7±1.3^{a}	0.0±0.0^{b}	0.0±0.0^{b}	0.1±0.2^{b}	**	**
Enterobacteriaceae or *Pseudomonadaceae*							
Escherichia-Shigella or *Pseudomonas*	8.7±9.5^{a}	0.4±0.6^{b}	0.3±0.5^{b}	0.7±1.3^{b}	0.3±0.4^{b}	***	**
Enterococcaceae							
Enterococcus	0.8±0.9^{a}	0.2±0.3^{ab}	0.6±1.1^{ab}	0.0±0.1^{b}	1.1±3.1^{ab}	**	**
Erysipelotrichaceae							
Turicibacter	0.0±0.0^{a}	0.1±0.2^{a}	1.7±1.9^{b}	0.9±1.4^{b}	1.8±2.9^{b}	***	***
Fusobacteriaceae							
Fusobacterium	0.3±0.6^{ab}	0.0±0.0^{a}	0.0±0.0^{a}	6.3±17.4^{b}	0.2±0.2^{b}	***	***
Helicobacteraceae							
Helicobacter	13.4±20.4^{a}	1.0±1.7^{b}	10.2±15.2^{ab}	13.3±16.9^{a}	11.7±18.5^{a}	**	*
Lachnospiraceae							
Lactobacillus	32.6±24.6^{ab}	33.0±34.8^{ab}	53.2±24.9^{a}	20.1±22.0^{ab}	11.7±12.3^{b}	*	*

Multi-affiliation	0.1±0.1^{ab}	1.3±1.9^{c}	0.1±0.1^{a}	0.1±0.1^{ab}	0.0±0.0^{b}	***	***
Micrococcaceae							
Rothia	0.6±0.7^{a}	0.1±0.1^{b}	0.7±0.7^{a}	0.0±0.0^{c}	0.0±0.0^{bc}	***	***
Peptostreptococcaceae							
Multi-affiliation *(Romboutsia* or unknown genus*)*	18.4±28.3^{ab}	6.7±22.3^{a}	4.9±5.9^{a}	44.3±31.8^{b}	38.7±30.3^{b}	***	***
Romboutsia	0.0±0.0^{a}	0.1±0.2^{a}	0.1±0.1^{a}	0.5±0.7^{ab}	11.8±18.9^{b}	***	**
Pseudomonadaceae							
Pseudomonas	0.0±0.0^{a}	0.0±0.0^{a}	0.0±0.0^{a}	0.1±0.4^{a}	0.6±0.7^{a}	*	*
Rhizobiaceae							
Allorhizobium-Neorhizobium-Pararhizobium-Rhizobium	0.0±0.0^{a}	0.0±0.0^{b}	0.5±0.4^{c}	0.0±0.0^{ab}	0.0±0.0^{a}	***	***
Rikenellaceae							
Alistipes	1.6±2.9^{a}	0.4±0.4^{a}	0.1±0.1^{b}	0.1±0.1^{b}	0.1±0.1^{b}	***	***
Ruminococcaceae							
Faecalibacterium	0.8±1.3^{a}	0.4±0.6^{a}	0.1±0.1^{b}	0.1±0.1^{b}	0.0±0.1^{b}	***	**
Negativibacillus	0.8±2.5	0.0±0.0	0.0±0.1	0.0±0.0	0.0±0.0	*	NS
Sphingomonadaceae							
Sphingomonas	1.6±1.5^{a}	14.3±11.4^{b}	6.6±8.2^{b}	2.6±4.8^{a}	6.8±9.7^{ab}	***	**
Streptococcaceae							
Streptococcus	1.7±2.2^{a}	3.6±11.4^{a}	2.8±4.3^{a}	0.1±0.1^{b}	0.1±0.3^{b}	***	***
Tannerellaceae							
Parabacteroides	0.8±1.9	0.0±0.0	0.0±0.0	0.0±0.0	0.0±0.0	NS	NS

Values are presented as Mean±SD.

Genera are sorted according to Family. Only genera with abundances above 0.5% in at least one experimental group are presented in the table.

C_{conv}: Control group before overfeeding, OF: Overfed group, C_{alt}: Control group before *Ad libitum* corn mixture feeding, NegSF: Negative response group to spontaneous fattening induction, PosSF: Positive response group to spontaneous fattening induction

Multi-affiliation: In FROGS, when performing taxonomic affiliation, if several blastn results have identical scores for an OTU, a taxonomy is determined for each hit at each taxonomic level. If these taxonomies differ across hits, the first level of conflict and all lower ones are set to 'Multi-affiliation.

* P<0.05, ** P<0.01, *** P<0.001, NS: Not Significant.

Means within a row with different superscripts differ significantly (p<0.05).

[1]P adjusted values according to the « false discory rate » (fdr) test.
